# Supplementary material for: An alternative pattern of head expansion during feeding in cichlids
Source: Commun Biol. 2025 Oct 9;8:1448. doi: 10.1038/s42003-025-08851-w (PMC12511344; doi:10.1038/s42003-025-08851-w)
Supplement: Supplementary file 2 — Supplementary Information [file 42003_2025_8851_MOESM2_ESM.pdf]

Supplementary Information for:

An alternative pattern of head expansion during feeding in cichlids

De Ridder, Jana[1,2]; Dujardin, Vincent[1]; Camacho Garcia, Julia[3]; Sawasawa, Wilson[3]; Aerts, Peter[1]; Svardal, Hannes[3,4]; Van Wassenbergh, Sam[1]

[1]Laboratory of Functional Morphology, Department of Biology, University of Antwerp, Antwerp, Belgium.

[2]Evolutionary Morphology of Vertebrates, Department of Biology, Ghent University, Ghent, Belgium

[3]Evolutionary Ecology Group, Department of Biology, University of Antwerp, Antwerp, Belgium.

[4]Vertebrate evolution, development and ecology, Naturalis Biodiversity Centre, Leiden, The Netherlands

Contact:
Sam Van Wassenbergh
Universiteitsplein 1
2610 Antwerpen
Belgium
sam.vanwassenbergh@uantwerpen.be
Tel: +32 3 265 1988

ORCID:
J.D.R.: 0009-0006-8833-8752
J.C.G.: 0000-0002-4828-1492
W.S.: 0000-0003-4997-7237
P.A.: 0000-0002-6867-5421
H.S.: 0000-0001-7866-7313
S.V.W: 0000-0001-5746-4621

Supplementary Table 1: head dimensions per tested individual. Head height is calculated as the distance between the top and bottom margin at the level of the pectoral fins on the lateral camera view. Length is the distance between the tip of the jaws and the middle between the points defining the height. Width is the distance between the left and right margin of the body at the level of the pectoral fins on the ventral camera view. Volume was calculated as a half ellipsoid (2/3 Π * length * width/2 * height/2).

ID	Length (mm)	Width (mm)	Height (mm)	Volume (mm^3)
Rhamphochromis sp. 'Chilingali'				
R1	31.5	11.0	18.1	3290
R2	29.5	9.6	16.2	2408
R3	31.5	10.7	18.1	3208
R4	29.8	10.7	17.9	2997
Chindongo saulosi				
C1	24.9	12.9	25.4	4259
C2	23.6	12.7	24.2	3780
Labeotropheus trewavasae				
L1	33.6	11.9	26.5	5532
L2	23.1	11.5	17.9	2494
L3	25.2	13.0	20.2	3470
L4	25.1	11.8	20.5	3160

Supplementary Table 2: results of linear mixed models testing the interaction between species and food type. Significant interactions are highlighted in green. A significance level of $p < 0.05$ is used.

	**F value**	**p-value**
Maximal mouth opening	$F_{2, 111}= 0.8691$	0.4222
Maximal hyoid depression	$F_{2, 112}= 0.9631$	0.3848
Timing maximal hyoid depression	$F_{2, 109}= 0.6939$	0.5018
Maximal premaxilla protrusion	$F_{2, 110}= 1.8575$	0.1609
Timing maximal premaxilla protrusion	$F_{2, 107}= 0.65$	0.5241
Maximal suspensorium abduction	$F_{2, 112}= 1.608$	0.2049
Timing maximal suspensorium abduction	$F_{2, 115}= 0.4736$	0.624
Maximal operculum abduction front	$F_{2, 112}= 0.1789$	0.8365
Timing maximal operculum abduction front	$F_{2, 112}= 1.7232$	0.1832
Maximal operculum abduction hind	$F_{2, 109}= 6.0931$	0.0031
Timing maximal operculum abduction hind	$F_{2, 112}= 0.8529$	0.4289

Supplementary Table 3: Linear mixed model results and pairwise comparisons between species and food type for models without a significant interaction between species and food type. For kinematic parameters indicated with a * normal linear models were used, due to a too small variability between individual fish. Significant results ($p < 0.05$) are highlighted in green. All variables were normalised to the cube root of the individual's head volume at rest.

		F or t value	p-value
Maximal mouth opening			
Food type		$F_{1, 112}$= 72.221	< 0.0001
Species		$F_{2, 5.71}$= 48.105	0.0003
Pairwise species	*L. trewavasae* - *C. saulosi*	$t_{6.23}$= -1.178	0.5053
	R. sp. 'Chilingali' - *L. trewavasae*	$t_{8.08}$= -8.994	< 0.0001
	R. sp. 'Chilingali' - *C. saulosi*	$t_{4.97}$= -7.105	0.0020
Maximal hyoid depression*			
Food type		F_1= 40.170	< 0.0001
Species		F_2= 54.423	< 0.0001
Pairwise species	*L. trewavasae* - *C. saulosi*	t_{114}= -0.360	0.9313
	R. sp. 'Chilingali' - *L. trewavasae*	t_{114}= -8.210	< 0.0001
	R. sp. 'Chilingali' - *C. saulosi*	t_{114}= -9.212	< 0.0001
Timing maximal hyoid depression			
Food type		$F_{1, 110}$= 0.0006	0.98001
Species		$F_{2, 4.61}$= 6.4253	0.04638
Pairwise species	*L. trewavasae* - *C. saulosi*	$t_{6.15}$= -2.649	0.0829
	R. sp. 'Chilingali' - *L. trewavasae*	$t_{10.8}$= 0.332	0.9413
	R. sp. 'Chilingali' - *C. saulosi*	$t_{3.75}$= 3.402	0.0628
Maximal premaxilla protrusion			
Food type		$F_{1, 111}$= 16.468	< 0.0001
Species		$F_{2, 4.87}$= 0.3285	0.7348
Pairwise species	*L. trewavasae* - *C. saulosi*	$t_{6.34}$= -0.597	0.8267
	R. sp. 'Chilingali' - *L. trewavasae*	$t_{11.9}$= -0.796	0.7126
	R. sp. 'Chilingali' - *C. saulosi*	$t_{3.60}$= -0.200	0.9784
Timing maximal premaxilla protrusion			
Food type		$F_{1, 109}$= 0.1078	0.7433
Species		$F_{2, 2.00}$= 2.198	0.3127
Pairwise species	*L. trewavasae* - *C. saulosi*	$t_{6.59}$= -1.463	0.3660
	R. sp. 'Chilingali' - *L. trewavasae*	$t_{13.0}$= -2.079	0.1330
	R. sp. 'Chilingali' - *C. saulosi*	$t_{3.50}$= -0.679	0.7889
Maximal suspensorium abduction			
Food type		$F_{1, 113}$= 16.422	< 0.0001
Species		$F_{2, 4.64}$= 115.034	0.0001
Pairwise species	*L. trewavasae* - *C. saulosi*	$t_{6.28}$= -1.999	0.1899
	R. sp. 'Chilingali' - *L. trewavasae*	$t_{10.2}$= -13.315	< 0.0001
	R. sp. 'Chilingali' - *C. saulosi*	$t_{3.94}$= -11.798	0.0007
Timing maximal suspensorium abduction*			
Food type		F_1= 0.00056	0.9404
Species		F_2= 8.6532	0.0003
Pairwise species	*L. trewavasae* - *C. saulosi*	t_{117}= -1.224	0.4415
	R. sp. 'Chilingali' - *L. trewavasae*	t_{117}= 2.220	0.0721
	R. sp. 'Chilingali' - *C. saulosi*	t_{117}= 4.108	0.0002
Maximal operculum abduction front			
Food type		$F_{1, 113}$= 91.478	< 0.0001
Species		$F_{2, 6.76}$= 59.187	< 0.0001
Pairwise species	*L. trewavasae* - *C. saulosi*	$t_{6.21}$= -0.526	0.8617
	R. sp. 'Chilingali' - *L. trewavasae*	$t_{8.17}$= -9.610	< 0.0001
	R. sp. 'Chilingali' - *C. saulosi*	$t_{4.89}$= -8.440	0.0010
Timing maximal operculum abduction front			

Food type		$F_{1, 114}$= 0.9131	0.3413
Species		$F_{2, 4.50}$= 11.535	0.01691
Pairwise species	*L. trewavasae* - *C. saulosi*	$t_{6.51}$= -1.141	0.5237
	R. sp. 'Chilingali' - *L. trewavasae*	$t_{11.5}$= 2.963	0.0307
	R. sp. 'Chilingali' - *C. saulosi*	$t_{3.68}$= 4.628	0.0254
Timing maximal operculum abduction hind*			
Food type		F_{1}= 11.800	0.0008
Species		F_{2}= 11.751	< 0.0001
Pairwise species	*L. trewavasae* - *C. saulosi*	t_{114}= -0.956	0.6059
	R. sp. 'Chilingali' - *L. trewavasae*	t_{114}= 3.012	0.0089
	R. sp. 'Chilingali' - *C. saulosi*	t_{114}= 4.700	< 0.0001

Supplementary Table 4: Pairwise comparisons between species and food type for models with a significant interaction between species and food type (shrimp "Sh" and spirulina ("Sp"). Above the diagonal t-values are given. Below the diagonal p-values are presented, with p < 0.05 as significance level (highlighted in green).

Maximal operculum abduction hind							
		R. sp. 'Chilingali'		*L. trewavasae*		*C. saulosi*	
		Sh	Sp	Sh	Sp	Sh	Sp
R. sp. 'Chilingali'	Sh		t_{108}= 6.341	$t_{30.0}$= -6.520	$t_{11.8}$= 8.003	$t_{7.67}$= -4.491	$t_{7.39}$= 6.209
	Sp	0.0001		$t_{21.5}$= -2.858	$t_{12.2}$= -3.750	$t_{7.90}$= -0.576	$t_{7.62}$= -2.245
L. trewavasae	Sh	< 0.0001	0.0856		t_{111}= 0.453	$t_{14.1}$= -2.130	$t_{13.8}$= -0.723
	Sp	< 0.0001	0.0253	0.9975		$t_{8.76}$= 2.798	$t_{8.48}$= -1.218
C. saulosi	Sh	0.0184	0.9900	0.3266	0.1481		t_{105}= 2.636
	Sp	0.0031	0.3174	0.9756	0.8180	0.0977	

Supplementary Figure 1:

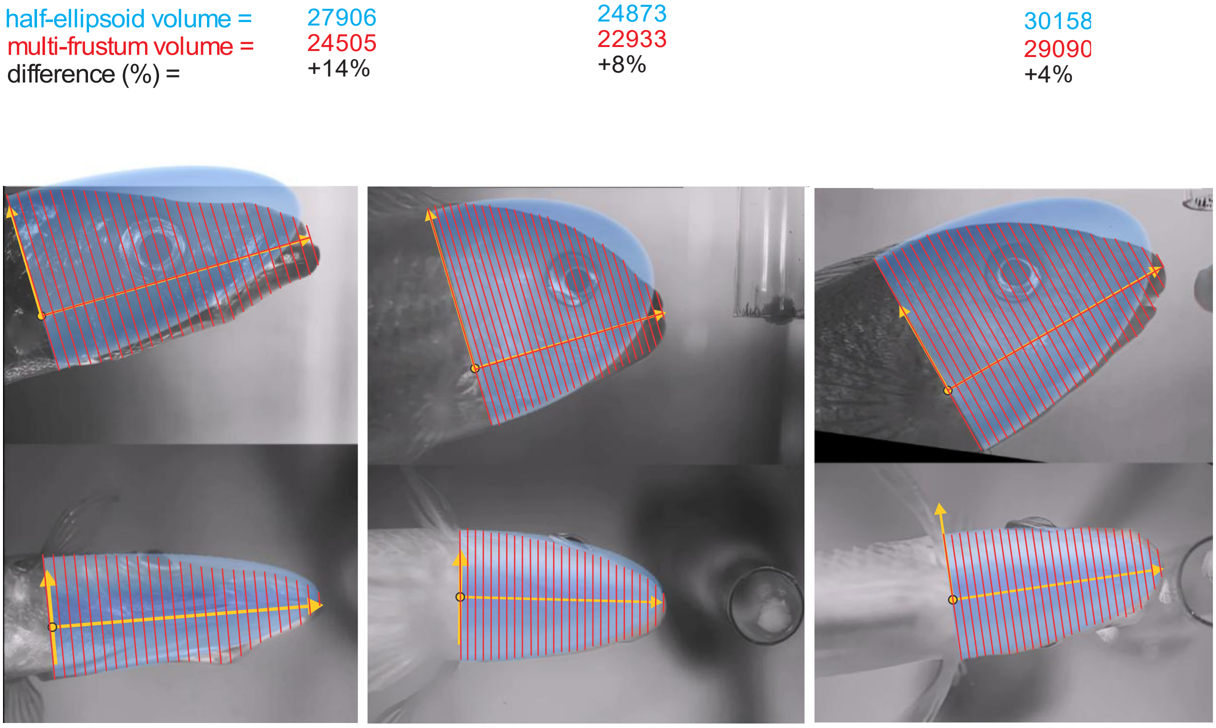

Supplementary Figure 1: Comparison between the half-ellipsoid method (blue overlay) and multi-frustum method (frustum axes in red) for estimating the initial volume of the head applied to one lateral and ventral image couple of each species studied (left: *Rhamphochromis* sp. 'Chilingali'; middle: *Chindongo saulosi*; right: *Labeotropheus trewavasae*). The numerical comparison, in arbitrary units, is given above. In our analyses, the half-ellipsoid method was used to normalise the head volume changes per individual (Supplementary Table 1). This analysis shows that the simpler of the two methods (i.e., half-ellipsoid) only slightly overestimates the head volume compared to the labour-intensive method (multi-frustum).

Supplementary Figure 2:

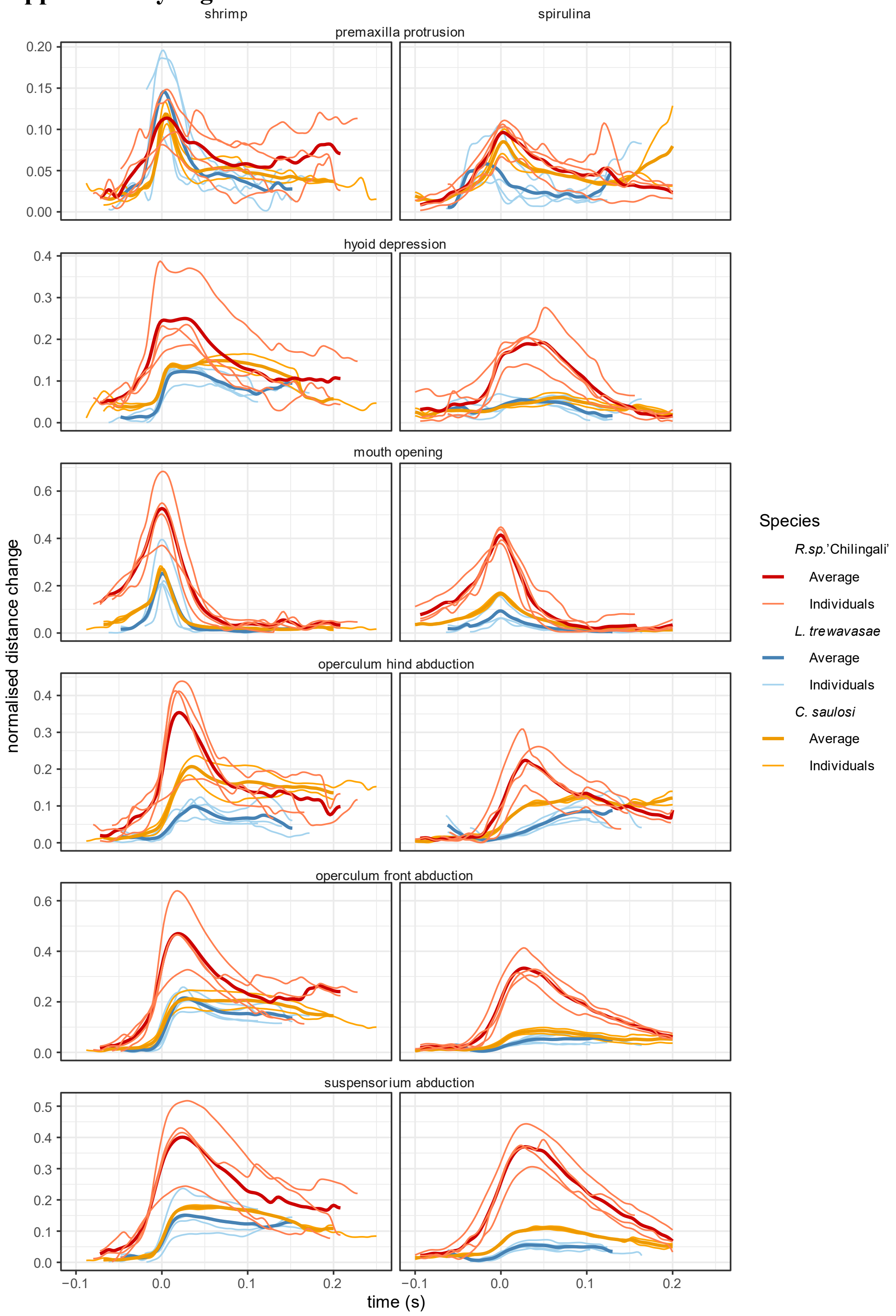

Supplementary Figure 2: Normalised distance change profiles per individual fish for the feeding experiment on shrimp (left column) and spirulina tablets (right column). Distances are normalised using the cube root of the individual's head volume. For every individual, the average over the different trials is shown. In bold, the average per species is added. Species are discerned according to the colour code legend on the right.
