# Supplementary material for: An alternative pattern of head expansion during feeding in cichlids
Source: Commun Biol. 2025 Oct 9;8:1448. doi: 10.1038/s42003-025-08851-w (PMC12511344; doi:10.1038/s42003-025-08851-w)
Supplement: Supplementary file 3 — Description of Additional Supplementary Files [file 42003_2025_8851_MOESM3_ESM.pdf]

Description of Additional Supplementary Files

File name: Supplementary Movie 1

Description: Slow-motions of high-speed videos of the three species studied on both experimental food types from lateral view (top) and ventral view (bottom).
