# Supplementary material for: An alternative pattern of head expansion during feeding in cichlids
Source: Commun Biol. 2025 Oct 9;8:1448. doi: 10.1038/s42003-025-08851-w (PMC12511344; doi:10.1038/s42003-025-08851-w)
Supplement: Supplementary file 5 — Reporting Summary [file 42003_2025_8851_MOESM5_ESM.pdf]

nature portfolio

Corresponding author(s): Sam Van Wassenbergh

Last updated by author(s): Aug 18, 2025

Reporting Summary

Nature Portfolio wishes to improve the reproducibility of the work that we publish. This form provides structure for consistency and transparency in reporting. For further information on Nature Portfolio policies, see our Editorial Policies and the Editorial Policy Checklist.

Statistics

For all statistical analyses, confirm that the following items are present in the figure legend, table legend, main text, or Methods section.

n/a	Confirmed	
☐	☒	The exact sample size (n) for each experimental group/condition, given as a discrete number and unit of measurement
☐	☒	A statement on whether measurements were taken from distinct samples or whether the same sample was measured repeatedly
☐	☒	The statistical test(s) used AND whether they are one- or two-sided<br>*Only common tests should be described solely by name; describe more complex techniques in the Methods section.*
☐	☒	A description of all covariates tested
☐	☒	A description of any assumptions or corrections, such as tests of normality and adjustment for multiple comparisons
☐	☒	A full description of the statistical parameters including central tendency (e.g. means) or other basic estimates (e.g. regression coefficient) AND variation (e.g. standard deviation) or associated estimates of uncertainty (e.g. confidence intervals)
☐	☒	For null hypothesis testing, the test statistic (e.g. F, t, r) with confidence intervals, effect sizes, degrees of freedom and P value noted<br>*Give P values as exact values whenever suitable.*
☒	☐	For Bayesian analysis, information on the choice of priors and Markov chain Monte Carlo settings
☒	☐	For hierarchical and complex designs, identification of the appropriate level for tests and full reporting of outcomes
☒	☐	Estimates of effect sizes (e.g. Cohen's d, Pearson's r), indicating how they were calculated

Our web collection on statistics for biologists contains articles on many of the points above.

Software and code

Policy information about availability of computer code

Data collection	Kinematic analysis: XMALab verson 2.1.0; CT-scan rendering: 3D Slicer version 5.3.0
Data analysis	Kinematic and volumetric data calculations Microsoft Excel version 365 (2024); Statistics: R version 4.0.3; Statistics R-packages: lmerTest package, version 3.1.3; pbkrtest package, version 0.5.1; emmeans package, version 1.7.4-1

For manuscripts utilizing custom algorithms or software that are central to the research but not yet described in published literature, software must be made available to editors and reviewers. We strongly encourage code deposition in a community repository (e.g. GitHub). See the Nature Portfolio guidelines for submitting code & software for further information.

Data

Policy information about availability of data

All manuscripts must include a data availability statement. This statement should provide the following information, where applicable:

- Accession codes, unique identifiers, or web links for publicly available datasets
- A description of any restrictions on data availability
- For clinical datasets or third party data, please ensure that the statement adheres to our policy

VAll raw experimental data, namely the video materials used in this study, as well as the numerical data for the main figures are available from Figshare: https://doi.org/10.6084/m9.figshare.25612023

Research involving human participants, their data, or biological material

Policy information about studies with human participants or human data. See also policy information about sex, gender (identity/presentation), and sexual orientation and race, ethnicity and racism.

Reporting on sex and gender	NA
Reporting on race, ethnicity, or other socially relevant groupings	NA
Population characteristics	NA
Recruitment	NA
Ethics oversight	NA

Note that full information on the approval of the study protocol must also be provided in the manuscript.

Field-specific reporting

Please select the one below that is the best fit for your research. If you are not sure, read the appropriate sections before making your selection.

☒ Life sciences ☐ Behavioural & social sciences ☐ Ecological, evolutionary & environmental sciences

For a reference copy of the document with all sections, see nature.com/documents/nr-reporting-summary-flat.pdf

Life sciences study design

All studies must disclose on these points even when the disclosure is negative.

Sample size	Video recordings were made from 4 R. sp. 'Chilingali', 4 L. trewavasae, and 2 C. saulosi individuals. The number of recordings differed between individuals and feeding experiment:<br>For R. sp. 'Chilingali' a total of 25 trials for the shrimp and 26 for the spirulina experiment were made (number of recordings per individual with IDs in subscript): shrimp: NR1= 4, NR2= 8, NR3= 6, NR4= 7; spirulina: NR1= 7, NR2= 6, NR3= 7, NR4= 6), for C. saulosi 21 and 23 trials (shrimp: NC1= 11, NC2= 10; spirulina: NC1= 12, NC2= 11) were used, and for L. trewavasae 8 and 18 trials (shrimp: NL1= 2, NL2= 2, NL3= 3, NL4= 1; spirulina: NL1= 6, NL2= 7, NL3= 2, NL4= 3). Due to the longer head of R. sp. 'Chilingali', some of the most posterior landmarks were not in the camera's view during the whole feeding act. In those cases, these landmarks were tracked for a fraction of the time or not used for the corresponding parameters. This reduced the sample size for some parameters (to NR2= 5 and NR4= 4 for hyoid depression and premaxilla protrusion in the shrimp experiment and to NR2= 5 and NR4= 4 for operculum abduction hind in de spirulina experiment.<br>A subset of the collected videos was used for head volume calculations:<br>For R. sp. 'Chilingali': spirulina and shrimp: NR1 = 1, NR2 = 1, NR3 = 1; for C. saulosi: spirulina: NC1 = 1, NC2 = 2, shrimp: NC1 = 2, NC 2 = 1; for L. trewavasae: spirulina: NL1 = 1, NL2 = 1, NL3 = 1, shrimp: NL1 = 1, NL3 = 1, NL4 = 1.
Data exclusions	None of the collected data were excluded from the analysis.
Replication	Replications were made in the form of samples from different individuals per species and feeding trials per individual (see 'Sample size').
Randomization	NA
Blinding	NA

Reporting for specific materials, systems and methods

We require information from authors about some types of materials, experimental systems and methods used in many studies. Here, indicate whether each material, system or method listed is relevant to your study. If you are not sure if a list item applies to your research, read the appropriate section before selecting a response.

Materials & experimental systems

n/a	Involved in the study
☒	☐ Antibodies
☒	☐ Eukaryotic cell lines
☒	☐ Palaeontology and archaeology
☐	☒ Animals and other organisms
☒	☐ Clinical data
☒	☐ Dual use research of concern
☒	☐ Plants

Methods

n/a	Involved in the study
☒	☐ ChIP-seq
☒	☐ Flow cytometry
☒	☐ MRI-based neuroimaging

Animals and other research organisms

Policy information about studies involving animals; ARRIVE guidelines recommended for reporting animal research, and Sex and Gender in Research

Laboratory animals	Rhamphochromis sp. 'Chilingali: offspring from a collection originating from Lake Chilingali from 2007.<br>Chindongo saulosi and Labeotropheus trewavasae: captive-bred individuals obtained from aquarium trade
Wild animals	NA
Reporting on sex	NA
Field-collected samples	NA
Ethics oversight	University of Antwerp Ethical Committee for Animal Testing file ECD-2019-02.

Note that full information on the approval of the study protocol must also be provided in the manuscript.

Plants

Seed stocks	NA
Novel plant genotypes	NA
Authentication	NA
